# The Mechanism of Melanocytes-Specific Cytotoxicity Induced by Phenol Compounds Having a Prooxidant Effect, relating to the Appearance of Leukoderma

**DOI:** 10.1155/2015/479798

**Published:** 2015-03-12

**Authors:** Takeshi Nagata, Shinobu Ito, Kazuyoshi Itoga, Hideko Kanazawa, Hitoshi Masaki

**Affiliations:** ^1^Institute of Advanced Biomedical Engineering and Science, Tokyo Women's Medical University, 2-10 Kawadacho, Shinjuku-ku, Tokyo 162-0054, Japan; ^2^I.T.O. Co. Ltd., 1-6-7-3F Naka-cho, Musashino, Tokyo 180-0006, Japan; ^3^Faculty of Pharmacy, Keio University, 1-5-30 Shibakoen, Minato-ku, Tokyo 105-8512, Japan; ^4^School of Bioscience and Biotechnology, Tokyo University of Technology, 1404-1 Katakuramachi, Hachioji, Tokyo 192-0982, Japan

## Abstract

Specific phenol compounds including rhododendrol (RD), a skin-brightening ingredient in cosmetics, are reported to induce leukoderma, inducing a social problem, and the elucidation of mechanism of leukoderma is strongly demanded. This study investigated the relationship among the cytotoxicities of six phenol compounds on B16F10 melanoma cells and HaCaT keratinocytes and generated reactive oxygen species (ROS). As a result, the cytotoxicity of RD on B16F10 cells was higher than that on HaCaT cells, and RD significantly increased intracellular ROS and hydrogen peroxide (H_2_O_2_) levels in B16F10 cells. Furthermore, although raspberry ketone (RK), RD derivative, also increased intracellular ROS in B16F10 cells, increase in ROS was suppressed by disodium dihydrogen ethylenediaminetetraacetate dehydrate (EDTA). The amounts of increased ROS with RK in HaCaT cells without melanocyte were further increased by tyrosinase. Therefore, tyrosinase, a metalloprotein having copper, was speculated to be one of causative agents allowing phenol compounds to work as a prooxidant. Hydroxyl radical was generated by adding a mixture of tyrosinase and H_2_O_2_ to RD, and the amount of the radical was further increased by UVB, indicating that RD cytotoxicity was caused by intracellularly increased ROS, which possibly related to phenol induced prooxidants.

## 1. Introduction

**Figure 1:**
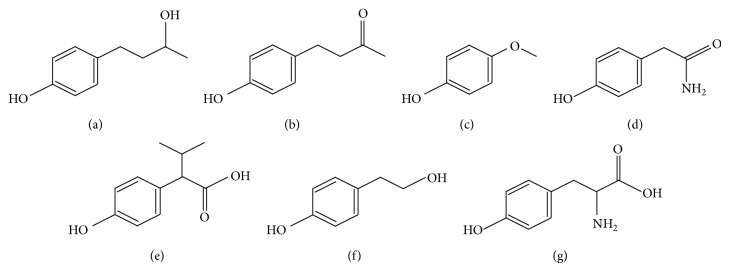
Chemical structures of phenolic compounds. (a) Rhododendrol (RD), (b) raspberry ketone (RK), (c) hydroquinone monomethyl ether (MEHQ), (d) p-hydroxyphenylacetamide (pHPA), (e) 2-(p-hydroxyphenyl)isovaleric acid (2pHP-IA), (f) p-hydroxyphenylethanol (pHPE), and (g) L-tyrosine.

In 2013, leukoderma has been reported to appear on the skin of women who use particular skin-brightening cosmetic products in Japan. The damage has been found widely not only in Japan but also in Asia, inducing a major social problem. More than 5,000 people have leukoderma in Japan at August 2013. Since skin-brightening cosmetic products containing a specific phenol compound, called rhododendrol (4-(4-hydroxyphenyl)-2-butanol (RD)) (Figure 1(a)), are reported to induce leukoderma, the elucidation of possible mechanism of leukoderma induced by RD is strongly demanded. Although leukoderma is reported to be induced by the death of melanocytes [1], RD is still unknown to show a specific cytotoxicity to melanocytes.

Melanocytes produce melanin actively as their characteristic features, and the cells have an indispensable enzyme, tyrosinase [2]. Tyrosinase is known to have copper in its active center [3–5]. Further, proteins containing copper are known to enhance oxidation reactions and induce apoptosis [6, 7]. Moreover, a metal chelating agent such as disodium dihydrogen ethylenediaminetetraacetate dehydrate (EDTA) inhibits the oxidation reaction of transition metals such as iron and copper with hydrogen peroxide (H_2_O_2_), which induces Fenton reaction [8, 9].

On the other hand, phenolic compounds including RD are used as building blocks for preparing various pharmaceutical compounds, cosmetic ingredients, and food additives because of their special characters in oxidation-reduction (redox) reactions. However, some phenolic compounds are known to show specific cytotoxicities to melanocytes. For instance, raspberry ketone (4-(4-hydroxyphenyl)-2-butanone (RK)) (Figure 1(b)), which is made by replacing the hydroxyl group of RD with ketone group, hydroquinone (HQ), hydroquinone monomethyl ether (MEHQ) (Figure 1(c)), 4-tertiary-butylphenol (4-TBP), 2-phenylphenol, and p-octylphenol have been known to cause leukoderma [10–16].

There are a few studies that directly detect the generation of reactive oxygen species (ROS) generated by metalloprotein* in vitro*. Actually, the authors have already confirmed that oxidation reactions promoted with iron proteins such as hemoglobin enhance the toxicity in ultraviolet- (UV-) ray-irradiation induced inflamed cutaneous tissues by comparing generated ROS quantitatively with electron spin resonance (ESR) spectroscopy [17].

Recently, the cytotoxicity of RD in melanocyte has been reported to be suppressed by phenylthiourea, a copper chelating agent, and the knockdown of tyrosinase [18]. This report indicates that the cytotoxicity of RD in melanocytes appears through the intermediary compounds of tyrosinase. Furthermore, the cytotoxicity of RD in melanocytes has been found to be involved in the oxidation of RD by tyrosinase [19]. For elucidating the cytotoxicity mechanism in melanocytes by RD, difference in the cytotoxicities between cells with and without melanin should be investigated.

This study, therefore, investigated the relationships among generated ROS and the cytotoxicities of RD in B16F10 melanoma cells and HaCaT keratinocytes having no melanin and compared the results to those of RK, MEHQ, p-hydroxyphenylacetamide (pHPA) (Figure 1(d)), 2-(p-hydroxyphenyl)isovaleric acid (2pHP-IA) (Figure 1(e)), and p-hydroxyphenylethanol (pHPE) (Figure 1(f)). This study also investigated whether RD could induce the generation of H_2_O_2_, which is known to induce directly the Fenton reaction in B16F10 cells. Hydroxyl radical (OH^∙^) was directly measured by ESR for confirming that the Fenton reaction was induced by H_2_O_2_ and copper with tyrosinase. Furthermore, the oxidation pathway of RD to catechol derivatives by tyrosinase was also investigated by high performance liquid chromatography (HPLC) analysis.

## 2. Methods

### 2.1. Materials

RK, pHPA, 2pHP-IA, pHPE, and H_2_O_2_ were purchased from Wako Pure Chemical Industries (Osaka, Japan). RD and EDTA were purchased from Nacalai Tesque (Kyoto, Japan). MEHQ was purchased from Tokyo Chemical Industry (Tokyo, Japan), respectively. Tyrosinase from mushroom, neutral red, and Triton X-100 reduced were purchased from Sigma-Aldrich (St. Louis, MO, USA), and 2′,7′-dichlorofluorescin diacetate (DCFDA) was purchased from EMD Millipore (Billerica, MA). Hoechst33342 was purchased from Life Technologies (Carlsbad, CA). 5,5-Dimethyl-1-pyrroline-N-oxide (DMPO) was purchased from Labotec (Tokyo). Dimethyl sulfoxide (DMSO) was purchased from Pierce Biotechnology (Rockford, IL). Ferrous sulfate was purchased from United States Pharmacopeial Convention (Rockville, MD). Dulbecco's modified Eagle medium (DMEM) was purchased from Nissui Pharmaceutical (Tokyo). Fetal bovine serum (FBS) was purchased from Biological Industries Israel (Kibbutz Beit-Haemek, Israel).

### 2.2. Cell Culture

B16F10 cells were cultured in DMEM containing 10% FBS at 37°C at a 5%-CO_2_ condition. HaCaT cells were cultured in DMEM containing 5% FBS at 37°C at a 5%-CO_2_ condition.

### 2.3. Cell Viability

B16F10 and HaCaT cells were cultured in DMEM containing 5.0 mM RD, RK, MEHQ, pHPA, 2pHP-IA, pHPE, or 10.0 mM MEHQ and pHPA individually, and for measuring the survival rates, the medium was replaced with DMEM containing 0.165 mg/mL neutral red. After being cultured for 2 h, the cells were washed with phosphate buffered saline (PBS), and neutral red incorporated in the cells was extracted with NR extraction solution, which was a mixture of methanol, acetic acid, and water at a ratio of 50 : 1 : 49. The absorbance of neutral red in a well was measured at 550 nm with a reference absorbance of 650 nm by a microplate reader (VMax Kinetic ELISA Microplate Reader) (Molecular Devices, Sunnyvale, CA). The cell survival rates were expressed as percent values of those of the cells incubated without test compounds described above.

### 2.4. Intracellular ROS Level

B16F10 cells were cultured in Hanks' balanced salt solution (HBSS) containing 1.0 mM RK, 20.0 *μ*M DCFDA, and 4.0 *μ*M Hoechst33342 for 30 min. After being incubated for 30 min, the cells were washed with HBSS and observed with a fluorescence microscope (EVOS FLoid Cell Imaging Station) (Life Technologies, Carlsbad, CA).

B16F10 and HaCaT cells were cultured in HBSS containing 1.0 mM RD, RK, MEHQ, pHPA, 2pHP-IA, or pHPE and 20.0 *μ*M DCFDA for 30 min, washed with HBSS, and solubilized with 0.5% Triton X-100 reduced. The fluorescence intensity of the medium containing the solubilized cells was measured at an exciting wavelength of 485 nm and an emission wavelength of 530 nm with a fluorescence microplate reader (Gemini EM fluorescence microplate reader) (Molecular Devices). The intracellular ROS level was expressed as a relative value, which was calculated from fluorescence intensity per 1.0 *μ*g protein of the control cells incubated without test samples.

The effects of tyrosinase and EDTA on change in intracellular ROS by exposure to RK were investigated by the following procedure. HaCaT cells were cultured in HBSS containing 50.0, 75.0, or 100.0 units/mL tyrosinase with 1.0 mM RK and 20.0 *μ*M DCFDA for 30 min. B16F10 cells were cultured in HBSS containing 0.25, 0.5, or 1.0 mM EDTA with 1.0 mM RK and 20.0 *μ*M DCFDA for 30 min. The fluorescence intensity of the medium was measured by the same method described above. The intracellular ROS level was expressed as a relative value, which was calculated from fluorescence intensity per 1.0 *μ*g protein of the control cells incubated without tyrosinase, EDTA, and RK.

### 2.5. Intracellular H_2_O_2_ Level

After being cultured in HBSS containing 5.0 mM RD or RK for 30 min, B16F10 cells were washed with HBSS and solubilized with 0.5% Triton X-100 reduced, and the intracellular amount of H_2_O_2_ was measured with Amplite fluorimetric hydrogen peroxide assay kit “Red Fluorescence” (ATT Bioquest, Sunnyvale, CA). The amount of H_2_O_2_ was expressed as a relative value, which was calculated from fluorescence intensity per 1.0 *μ*g protein of the control cells incubated without test samples such as RD and RK.

### 2.6. ESR Signal Measurement

A JES-FA200 ESR spectrometry (JEOL, Tokyo) was used for ESR signal measurement. ESR spectrometry conditions used for estimating radical species with adequate spin-trap reagents were as follows: microwave frequency, 9414.499 ± 5.000 MHz; microwave power, 4.00 mW; field center, 335.32 ± 0.5 mT; sweep width, ±5.00 mT; modulation frequency, 100.00 kHz; sweep time, 0.5~5 min; amplitude, 1.500~2.500; and time constant, 0.03~0.5 s, at room temperature. This study used an ESR universal cavity (ES-UCX2: TE011-mode cavity) (JEOL) with X-band microwave units (8.750–9.650 GHz), an aqueous sample cell (ES-LC12, JEOL) with a sample volume of 20~100 *μ*L, and a quartz cell (Labotec) with a home-made cover glass (40 × 5 × 0.5 mm). As an ESR standard marker, manganese oxide (MnO) powder (MO7-FB-4, JEOL) was used. As a spin-trap agent, 5,5-dimethyl-1-pyrroline-N-oxide (DMPO, 100 w/w% liquid) and 10 w/w% DMSO solution were used. The following test samples were solved in 0.1 M PBS (pH 7.5). RD (100 *μ*M) was added to 100 units/mL tyrosinase. DMPO (20 *μ*L) was added to the sample solutions (20 *μ*L) immediately, and the radical signals were measured by ESR. UVB (290~340 nm) irradiation was introduced to samples at a dose of 200~1000 J/m^2^ by a USHIO Optical Modulex (Ushio, Tokyo). To identify observed peaks, signals were analyzed by specialized ESR analysis software (A-System vl.40 ISAJ, FA-manager vl.20) (JES, Tokyo), which was installed in the ESR device, and allowed to determine *g*-value and calculated hfcc from the distance between peaks. To identify ESR signals, the *g*-value and hfcc of the measurable peaks were compared with the peak values of the standard OH^∙^. The standard OH^∙^ was generated by the reaction of ferrous sulfate solution (100 nM) and H_2_O_2_ (30 mM).

### 2.7. HPLC Analysis

After 100.0 *μ*M RD was added to 100.0 units/mL tyrosinase, the sample mixture was incubated for 0, 1.5, and 30 min at 37°C and was injected into an HPLC instrument. Sample (50.0 *μ*L) was separated on an octadecylsilane (ODS) reverse-phase column (Shodex C18P-4E column) (Showa Denko, Tokyo) at 40°C. The mobile phase, which was a mixture of acetonitrile and 0.03 M KH_2_PO_4_ at a ratio of 60 : 40, was used at a flow rate of 0.7 mL/min. The HPLC system consisted of a SCL-10A system controller, a LC-10AD pump, a SPD-10A UV/VIS detector, a CTO-10AC column oven, and a SIL-10A injector (Shimadzu, Kyoto). The separated peaks were monitored at 300 nm with the detector.

### 2.8. Protein Assay

Protein concentration was determined with bicinchoninic acid- (BCA-) protein assay kit (Pierce Biotechnology).

### 2.9. Statistical Analysis

Comparison between two groups was performed by Student's* t*-test, and the probability less than 0.05 (*P* < 0.05) was considered statistically significant. Correlations were analyzed by calculating Pearson correlation coefficients (*r*).

## 3. Results

### 3.1. The Cytotoxicities of Phenol Compounds to B16F10 and HaCaT Cells

**Figure 2:**
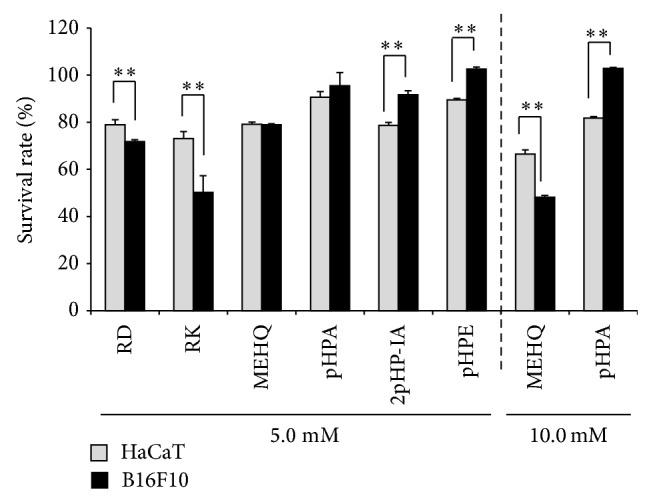
Effects of phenol compounds on the survival rates of B16F10 melanoma cells and HaCaT keratinocytes. B16F10 and HaCaT cells were treated with 5.0 mM rhododendrol (RD), raspberry ketone (RK), hydroquinone monomethyl ether (MEHQ), p-hydroxyphenylacetamide (pHPA), 2-(p-hydroxyphenyl)isovaleric acid (2pHP-IA), p-hydroxyphenylethanol (pHPE), 10.0 mM MEHQ, or pHPA for 24 h. Cell survival rates were determined by using neutral red. The black and gray columns indicate the averaged survival rates of B16F10 and HaCaT cells, respectively. The lines on the columns show the standard deviations (*n* = 5). Two asterisks (∗∗) indicate that the probabilities of significance levels are less than 0.01 (*P* < 0.01).

Figure 2 shows the survival rates of B16F10 and HaCaT cells treated with RD, RK, MEHQ, pHPA, 2pHP-IA, or pHPE for 24 h. The survival rates of HaCaT cells treated with 5.0 mM RD and RK were found to be significantly higher than those of B16F10 cells. The survival rates of HaCaT cells treated with 10.0 mM MEHQ were found to be significantly higher than those of B16F10 cells. The results indicated that RD, RK, and MEHQ had a specific cytotoxicity to B16F10 cells. However, the survival rates of B16F10 cells treated with 5.0 mM 2pHP-IA, pHPE, and 10.0 mM pHPA were found to be significantly higher than those of HaCaT cells, indicating that 2pHP-IA, pHPE, and pHPA showed no B16F10 cells-specific cytotoxicity.

### 3.2. Effects of Phenol Compounds on the Intracellular ROS Levels of B16F10 and HaCaT Cells

**Figure 3:**
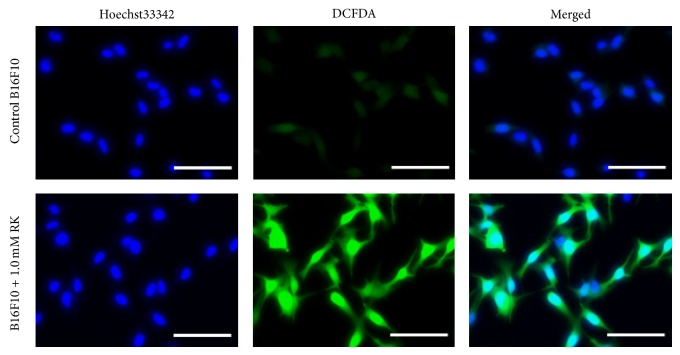
The fluorescence microphotographs of intracellular reactive oxygen species (ROS) level in B16F10 melanoma cells treated with raspberry ketone (RK). After being treated with 1.0 mM RK, 20.0 *μ*M 2′,7′-dichlorofluorescin diacetate (DCFDA), and 4.0 *μ*M Hoechst33342 for 30 min, B16F10 cells were observed with a fluorescence microscope. DCFDA and Hoechst33342 were able to show ROS level and the nuclei of the cells, respectively. Scale bar, 100 *μ*m.

**Figure 4:**
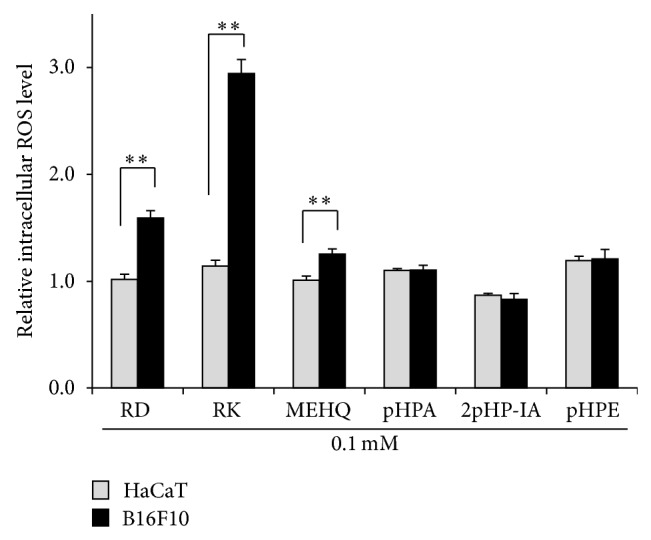
Effects of phenol compounds on the intracellular reactive oxygen species (ROS) level of B16F10 melanoma cells and HaCaT keratinocytes. After B16F10 and HaCaT cells were treated with 1.0 mM rhododendrol (RD), raspberry ketone (RK), hydroquinone monomethyl ether (MEHQ), p-hydroxyphenylacetamide (pHPA), 2-(p-hydroxyphenyl)isovaleric acid (2pHP-IA), or p-hydroxyphenylethanol (pHPE), and 20.0 *μ*M 2′,7′-dichlorofluorescin diacetate (DCFDA) for 30 min, the fluoresce intensities were measured. The black and gray columns indicate the averaged intracellular ROS levels in B16F10 and HaCaT cells, respectively. The lines on the columns show the standard deviations (*n* = 5). One (∗) and two (∗∗) asterisks indicate that the probabilities of significance levels are less than 0.05 (*P* < 0.05) and 0.01 (*P* < 0.01), respectively.

**Figure 5:**
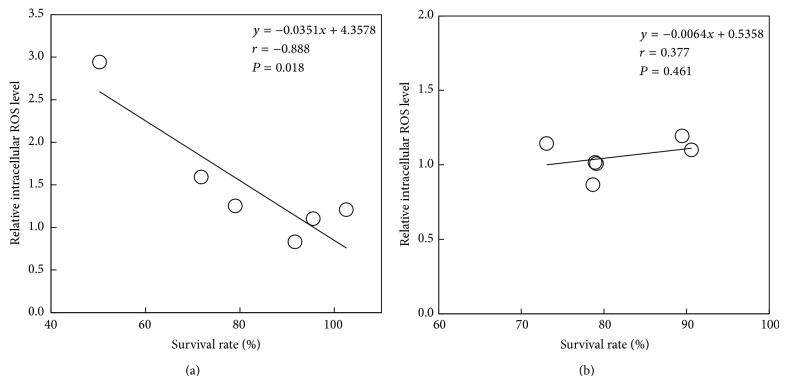
Relationships among the cell viabilities of (a) B16F10 melanoma cells and (b) HaCaT keratinocytes, which were treated with 5.0 mM rhododendrol (RD), raspberry ketone (RK), hydroquinone monomethyl ether (MEHQ), p-hydroxyphenylacetamide (pHPA), 2-(p-hydroxyphenyl)isovaleric acid (2pHP-IA), or p-hydroxyphenylethanol (pHPE), and the generated amounts of ROS in both cells, which were treated with 1.0 mM of the phenolic compounds. Pearson correlation coefficients (*r*) were calculated, and the probability less than 0.05 (*P* < 0.05) was considered statistically significant.

After B16F10 cells were treated with 1.0 mM RK, which is made by replacing the hydroxyl group of RD with ketone group, for 30 min, the generation of ROS was clearly confirmed in the cells (Figure 3). Since the intensity of fluorescence emitted from B16F10 cells treated with 5.0 mM RK was too high to be measured with the instrument for measuring intracellular ROS level, RK concentration was reduced from 5.0 to 1.0 mM and the experiment which used B16F10 cells treated with 0.1 mM RK was performed. Similarly, after B16F10 and HaCaT cells were treated with phenolic compounds including 1.0 mM RD, RK, MEHQ, pHPA, 2pHP-IA, or pHPE for 30 min, the generated amounts of ROS in the cells were determined, and only by the treatments with RD, RK, and MEHQ, the increased amounts of ROS in B16F10 cells were found to be higher than those of HaCaT cells (Figure 4). The correlations between the cell viabilities of B16F10 and HaCaT cells, which were treated with 5.0 mM RD, RK, MEHQ, pHPA, 2pHP-IA, and pHPE, and the generated amounts of ROS in both cells, which were treated with 1.0 mM of the phenolic compounds, were investigated (Figure 5). Although a significant negative correlation was found between the cell viabilities and the generated amounts of ROS in B16F10 cells (Figure 5(a)), no significant correlation was confirmed in HaCaT cells (Figure 5(b)), indicating that only B16F10 cells showed decrease in cell viability with increasing the generated amounts of ROS.

### 3.3. Effect of Tyrosinase on Intracellular ROS Induced by RK

**Figure 6:**
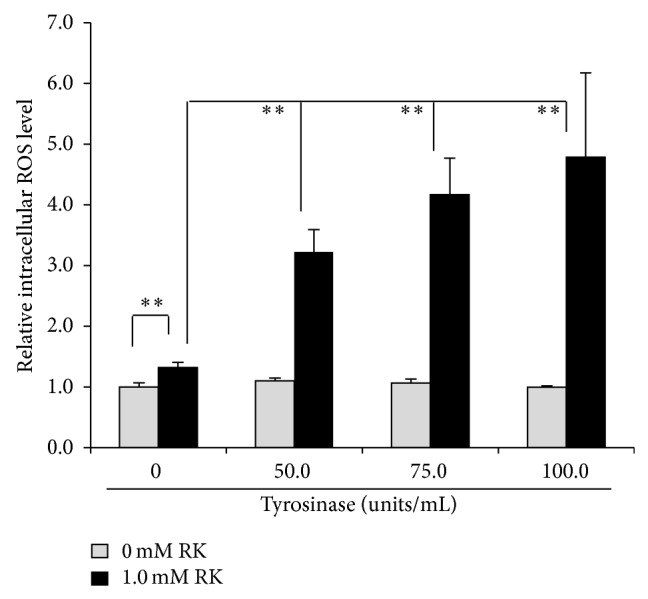
Effect of tyrosinase on intracellular reactive oxygen species (ROS) induced by raspberry ketone (RK). After HaCaT keratinocytes were treated with 50.0~100.0 units/mL tyrosinase, 1.0 mM RK, and 20.0 *μ*M 2′,7′-dichlorofluorescin diacetate (DCFDA) for 30 min, the fluoresce intensities were measured. The black and gray columns indicate the averaged intracellular ROS levels in B16F10 treated with 0 and 1.0 mM RK, respectively. The lines on the columns show the standard deviations (*n* = 5). Two asterisks (∗∗) indicate that the probabilities of significant levels are less than 0.01 (*P* < 0.01).

Tyrosinase was investigated to be involved in the increase in intracellular ROS level. As a result, 50.0, 75.0, and 100.0 units/mL tyrosinase were confirmed to further promote increase in the intracellular ROS level in HaCaT cells treated with 1.0 mM RK (Figure 6).

### 3.4. Effect of EDTA on Intracellular ROS Induced by RK

**Figure 7:**
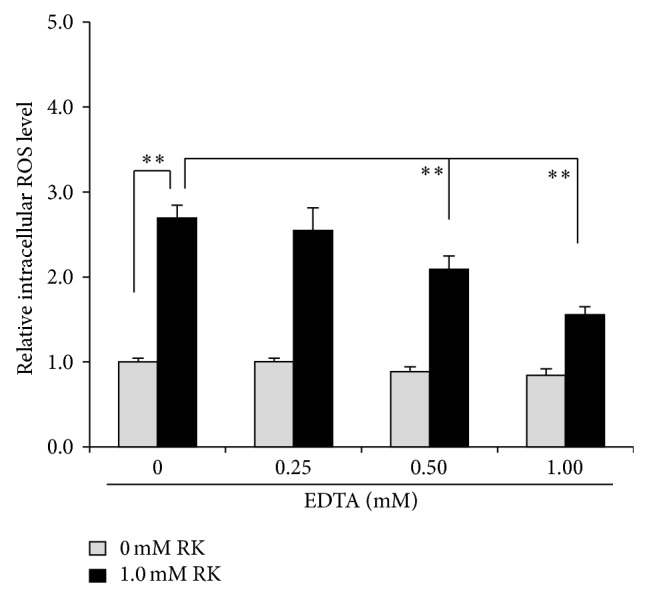
Effect of disodium dihydrogen ethylenediaminetetraacetate dihydrate (EDTA) on intracellular reactive oxygen species (ROS) induced by raspberry ketone (RK). After B16F10 melanoma cells were treated with 0.25~1.0 mM EDTA, 1.0 mM RK, and 20.0 *μ*M 2′,7′-dichlorofluorescin diacetate (DCFDA) for 30 min, the fluoresce intensities were measured. The black and gray columns indicate the averaged intracellular ROS levels in B16F10 treated with 0 and 1.0 mM RK, respectively. The lines on the columns show the standard deviations (*n* = 5). Two asterisks (∗∗) indicate that the probabilities of significant levels are less than 0.01 (*P* < 0.01).

EDTA, a metal chelating agent, was investigated to decrease ROS generated in B16F10 cells with RK intracellularly. As a result, 0.5 and 1.0 mM EDTA were confirmed to reduce the intracellular ROS level increased with 1.0 mM RK (Figure 7).

### 3.5. Effect of RD on the Intracellular H_2_O_2_ of B16F10 Cells

**Figure 8:**
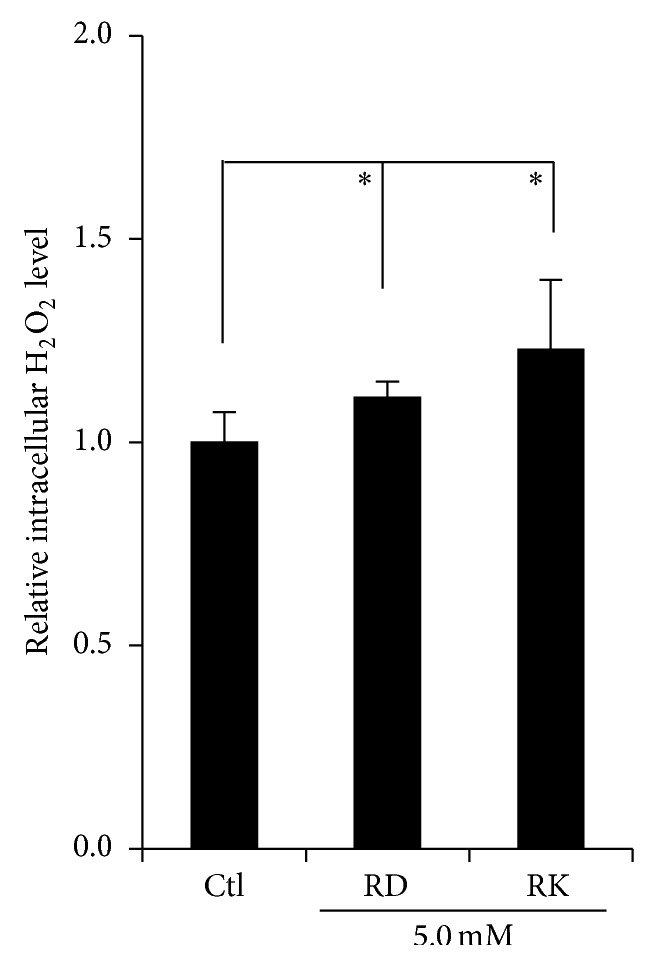
Effect of rhododendrol (RD) on the intracellular hydrogen peroxide (H_2_O_2_) level of B16F10 melanoma cells. After B16F10 cells were treated with 5.0 mM RD and raspberry ketone (RK) for 15 min, the intracellular H_2_O_2_ level of the cells was measured by Amplite fluorimetric hydrogen peroxide assay kit “Red Fluorescence.” The black columns indicate the averaged intracellular H_2_O_2_ levels in B16F10 treated with 5.0 mM RD and RK. The lines on the columns show the standard deviations (*n* = 5). One (∗) and two (∗∗) asterisks indicate that the probabilities of significance levels are less than 0.05 (*P* < 0.05) and 0.01 (*P* < 0.01), respectively.

B16F10 cells were treated with RD and RK, and the intracellular H_2_O_2_ level in the cells was measured. As a result, 5.0 mM RD and RK were found to increase the H_2_O_2_ level significantly (Figure 8).

### 3.6. ESR Experiment

**Figure 9:**
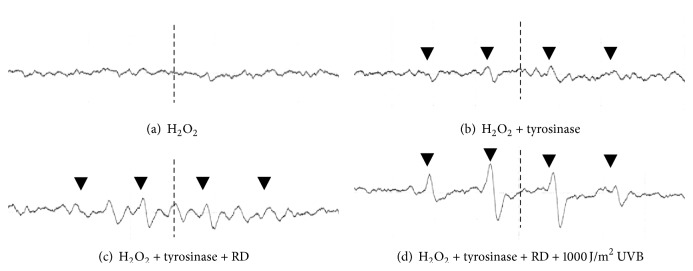
Electron spin resonance (ESR) spectra of 5,5-dimethyl-1-pyrroline-N-oxide (DMPO) adduct signals in a sample mixture of hydrogen peroxide (H_2_O_2_), tyrosinase, and rhododendrol (RD) irradiated with ultraviolet B (UVB) at a dose of 1000 J/m^2^. After 100 *μ*M RD was added to 100 units/mL tyrosinase, DMPO (20 *μ*L) was added to the sample mixture (20 *μ*L), which was irradiated with UVB immediately, and the radical signals were measured by ESR. (a) H_2_O_2_, (b) a mixture of H_2_O_2_ and tyrosinase, (c) a mixture of H_2_O_2_, tyrosinase, and RD, and (d) a mixture of H_2_O_2_, tyrosinase, and RD, and the mixture was irradiated with UVB.

**Figure 10:**
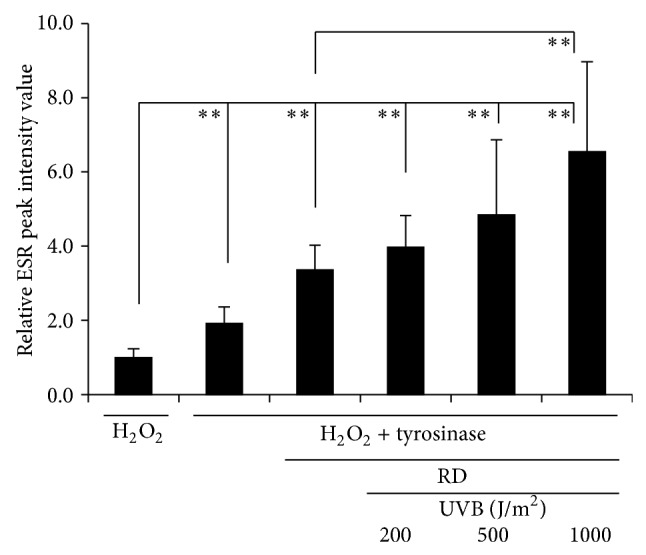
Relative electron spin resonance (ESR) peak intensity values of 5,5-dimethyl-1-pyrroline-N-oxide (DMPO) adduct signals in a sample mixture of hydrogen peroxide (H_2_O_2_), tyrosinase, and rhododendrol (RD), and the mixture was irradiated with ultraviolet B (UVB). After 100 *μ*M RD was added to 100 units/mL tyrosinase, DMPO (20 *μ*L) was added to the sample mixture (20 *μ*L), which was irradiated with UVB (200~1000 J/m^2^) immediately, and the radical signals were measured by ESR. The black columns indicate the averaged ESR peak intensity. The lines on the columns show the standard deviations (*n* = 8). Two asterisks (∗∗) indicate that the probabilities of significant levels are less than 0.01 (*P* < 0.01).

After RD was added to a sample mixture of tyrosinase and H_2_O_2_ and irradiated by UVB at a dose of 1000 J/m^2^, DPMO-radical adduct spectra of the samples were detected by ESR (Figure 9). The heights of ESR peaks, which are marked with downward arrow heads (▼), were found to increase with RD (Figure 9(c)) and further increase by UVB irradiation (Figure 9(d)). These four waveforms were found to be derived from hydroxyl radical (OH^∙^) by their *g*-values and hfcc values in the measurable peaks in ESR.  Figure 10 indicates the relative values of the peak intensities in the secondary waves of OH^∙^ spectra. The samples were irradiated with UVB at 200, 500, and 1000 J/m^2^, and the peak intensity of OH^∙^ spectra of a sample mixture of tyrosinase, H_2_O_2_, and RD was significantly increased at a dose of 1000 J/m^2^. Further, the peak intensity of OH^∙^ spectra was found to increase with UVB irradiation dose-dependently.

### 3.7. HPLC Experiment

**Figure 11:**
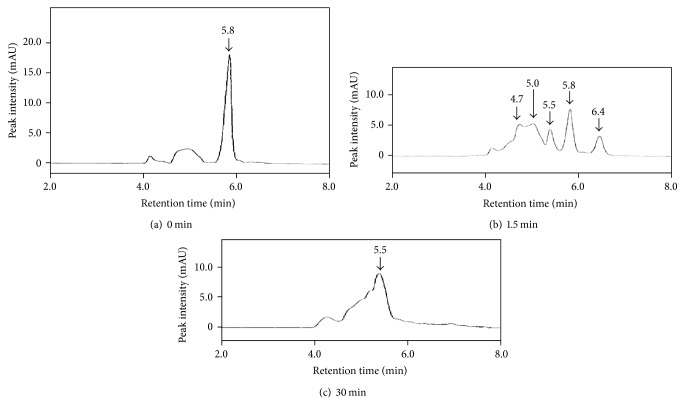
High performance liquid chromatography (HPLC) analysis of rhododendrol (RD). After 100.0 *μ*M RD was added to 100.0 units/mL tyrosinase, the sample mixture was incubated for (a) 0, (b) 1.5, and (c) 30 min at 37°C and injected into the reverse-phase HPLC. The separated peaks were monitored at 310 nm.

After 100.0 *μ*M RD was added to 100.0 units/mL tyrosinase, the sample mixture was incubated for 1.5 or 30 min at 37°C and was analyzed by HPLC. The separated peaks were monitored at 310 nm. Although the sample gave a peak derived from RD at a retention time (RT) of 5.8 mm (Figure 11(a)), a 1.5-minute incubation also gave unknown peaks at 4.7, 5.0, 5.5, and 6.4 min (Figure 11(b)). On the contrary, a 30-minute incubation gave only an unknown peak at 5.5 min, which was found to be different from the RD peak (Figure 11(c)). Since a mixture of acetonitrile and 0.03 M KH_2_PO_4_ at a ratio of 60 : 40 was used as a mobile phase in the reverse-phase HPLC column for analyzing the samples, observed RT was expected to be slower for higher hydrophobic substances. Therefore, unknown peaks found at earlier than 5.8 min were thought to be higher water-soluble substances than RD. Also, an unknown peak at 6.4 min, which was obtained from RD by being reacted with tyrosinase for 1.5 min (Figure 11(b)), was found to be identical with RK peak (data not shown).

## 4. Discussion

RK and MEHQ are reported to show their cytotoxicities more strongly in B16 cells than human fibrosarcoma HT1080 cells [20]. However, there is no study investigating the melanocyte-specific cytotoxicities of other phenolic compounds including RD, pHPA, 2pHP-IA, and pHPE. This study evaluated the melanocyte-specific cytotoxicities of phenolic compounds by investigating the effects of RD, RK, MEHQ, pHPA, 2pHP-IA, and pHPE on the survival rates against B16F10 and HaCaT cells. As a result, RD, RK, and MEHQ, which are already reported to cause leukoderma on the skin, were found to reduce the survival rate of B16F10 cells more strongly than that of HaCaT cells, and the B16F10 cells-specific cytotoxicities of these phenolic compounds were confirmed (Figure 2), indicating that the cause of leukoderma in the skin due to RD was speculated to be a melanocytes-specific cytotoxicity, which was similar to those of RK and MEHQ.

The productions of ROS inside melanocytes could possibly contribute to the appearance of the melanocytes-specific cytotoxicity, because normal melanocytes treated with 4-TBP, which shows a melanocytes-specific cytotoxicity, for 30 min are reported to increase the intracellular amounts of ROS [21]. Moreover, the amounts of ROS in the melanocytes of the patients with leukoderma on the skin are reported to be higher than those of normal melanocytes of healthy people [22]. Naturally, this study investigated changes in the intracellular amounts of ROS in B16F10 and HaCaT cells that were treated with RD, RK, MEHQ, pHPA, 2pHP-IA, or pHPE and observed that the amounts of ROS in B16F10 cells treated with RD, RK, and MEHQ, which are already reported to give leukoderma on the skin, were increased significantly compared to those of HaCaT cells (Figure 4). Remarkably, RK was found to increase the intracellular amount of ROS more in B16F10 than HaCaT cells by 157.4%. These results showed that the B16F10 cells-specific cytotoxicities of phenolic compounds including RD, RK, and MEHQ were speculated to be originated from the increased amounts of ROS in B16F10 cells.

All phenolic compounds used in this study were examined for their cytotoxicities and ROS productions in B16F10 and HaCaT cells (Figures 2 and 4), and only RD, RK, and MEHQ showed a higher cytotoxicity and a higher ROS generating ability in B16F10 than HaCaT cells, suggesting that other phenolic compounds were unnecessary to be tested in further experiments including that with tyrosinase. In spite of their comparable B16F10 cell-specific cytotoxicities and ROS generating abilities (Figures 2 and 4), the chemical structure of RK is similar to that of RD, an approved cosmetic ingredient, but that of MEHQ is different from them (Figure 1). Therefore, only RD and RK were used in further experiments.

Melanocytes including B16F10 cells are known to produce melanin for various physiological purposes, and the production process is called melanogenesis. For performing melanogenesis actively, tyrosinase is known as an indispensable enzyme and has copper in its active center [3–5]. Transition metals including iron and copper are known to react with redox molecules with generating ROS [23]. Since phenolic compounds including RD have a chemical structure similar to that of L-tyrosine (Figure 1(g)), it may act as a substrate for tyrosinase. Actually, 4-TBP binds to the active center of the enzyme and works as a competitive inhibitor [24]. By considering the structures of these phenolic compounds, this study assumed that the distance between the terminal oxygen atom of the side chain and the benzene ring of phenolic compounds was important. The distances in RD and RK having a B16F10 cells-specific cytotoxicity are speculated to be approximately 0.5 nm, and this distance was assumed to be substantially the same as that of L-tyrosine. This distance was speculated to be related to the depth of the pocket of the active center in tyrosinase. Therefore, the phenolic compounds having the same distance as that of L-tyrosine could be easily oxidized by tyrosinase.

The reaction of RD or RK with copper in tyrosinase has a possibility to induce the enhancement of oxidation, which can generate reactive oxygen species (ROS), resulting in the possible appearance of cytotoxicity eventually by the prooxidant effects of RD and RK. Since (1) 50.0~100.0 units/mL tyrosinase further increased the intracellular amount of ROS, which had been increased by 1.0 mM RK, in HaCaT cells by 144.2~262.5% significantly (Figure 6), and (2) 0.5 and 1.0 mM EDTA, a chelating agent, significantly suppressed the intracellular amount of ROS, which had been increased by 1.0 mM RK, in B16F10 cells by approximately 22.3 and 42.2%, respectively (Figure 7), copper in tyrosinase was speculated to be involved in generating ROS. Therefore, copper in tyrosinase might contribute to the increase in the amount of ROS generated by RD in B16F10 cells. Reaction of transition metals such as iron and copper with H_2_O_2_ is reported to induce Fenton reaction, which is known as a reaction generating highly and fatally cytotoxic ROS such as OH^∙^ [25–34]. In the authors' previous study, iron proteins such as hemoglobin in the cutaneous tissue and ascorbic acid (AA) are reported to promote Fenton reaction, which enhances UV-irradiation induced cytotoxicity [17]. Reaction of copper and AA is reported to promote Fenton reaction [23]. Since the intracellular amount of H_2_O_2_ in B16F10 cells was significantly increased by RD by approximately 11.0% (Figure 8), RD also had a possibility to induce Fenton reaction with copper in tyrosinase. As shown in Figures 9 and 10, OH^∙^ was found to be generated by the reaction of RD and tyrosinase, and OH^∙^ might be a cause of the B16F10 cells-specific cytotoxicity of RD. In Figure 9(c), specific 4 peaks derived from OH^∙^ were confirmed by ESR. Furthermore, OH^∙^ generated by the reaction of RD and tyrosinase was found to be increased by UVB irradiation dose-dependently (Figure 10), and the increase agreed with the fact that RD-induced leukoderma appears more strongly on UV-exposed sites such as face, neck, and hands.

The substrates of tyrosinase are L-tyrosine, L-dopa, and 5,6-dihydroxyindole (DHI) [2]. L-tyrosine is oxidized to L-dopa, a catechol derivative, by tyrosinase, and L-dopa is also oxidized to dopaquinone, a quinone derivative, by tyrosinase. Having a structure similar to that of L-tyrosine, 4-TBP is reported to be able to bind with the active center of tyrosinase and work as an inhibitor [24]. Having a structure more similar to that of L-tyrosine than that of 4-TBP, RD was speculated to bind with the active center of tyrosinase. Therefore, RD, as well as L-tyrosine, was speculated to be oxidized to catechol and quinone derivatives by tyrosinase. Actually, 4-TBP and 4-tertiary-butyl catechol (4-TBC) are reported to be converted into quinone derivatives by tyrosinase [35]. HPLC analysis showed that two substances at 4.7 and 5.0 min, which were slower than RD peak at 5.8 min, were found to be generated by the reaction of RD and tyrosinase for 1.5 min (Figure 11(b)). As the property of reverse-phase HPLC column, RT is known to be inversely proportional to the number of hydroxyl groups. Therefore, two substances which appeared at 4.7 and 5.0 min, which were made from RD by reacting with tyrosinase for 1.5 min, were speculated to have more hydroxyl groups than RD. A substance at 6.4 min was also found to be generated by the reaction of RD and tyrosinase for 1.5 min (Figure 11(b)) and might be derived from RK, because the RT of this peak agreed with that of RK. Since RD is made by adding a hydrogen atom to RK structure, RK was speculated to be generated by the oxidization of RD by tyrosinase. Furthermore, when RD reacted with tyrosinase for 30 min, a substance at 5.5 min was found to be generated (Figure 11(c)). This substance was speculated to have fewer hydroxyl groups than two substances made from RD by reacting with tyrosinase for 1.5 min. These results suggested that RD might be converted into a substance at 5.5 min through two intermediated substances appearing at 4.7 and 5.0 min.

**Figure 12:**
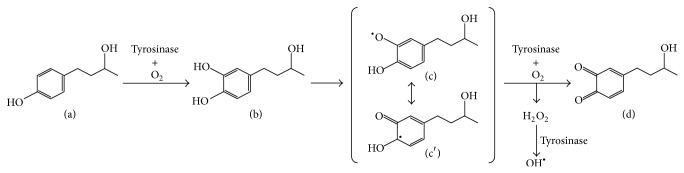
A proposed reactive oxygen species (ROS) generation mechanism in B16F10 melanoma cells by rhododendrol (RD). (a) RD, (b) RD-catechol, (c) RD-semiquinone, and (d) RD-quinone.

Figure 12 shows a possible pathway where RD was eventually converted into a quinone derivative through catechol and semiquinone derivatives by tyrosinase. A catechol derivative of RD (RD-catechol, Figure 12(b)), two semiquinone derivatives of RD (RD-semiquinone) (Figures 12(c) and 12(c′)), which are prooxidant molecules of RD, and a quinone derivative of RD (RD-quinone) (Figure 12(d)) were arranged in the descending order of the number of hydroxyl groups; RD-catechol > RD-semiquinone > RD-quinone. When these three substances were assumed to be three substances appearing at 4.7, 5.0, and 5.5 min (Figures 11(b) and 11(c)), the results of reverse-phase HPLC analysis (Figure 11) were able to support a possible pathway of oxidization of RD quite logically (Figure 12). Because the oxidation of RD to its quinone derivatives was speculated to be performed more possibly by intracellular tyrosinase than extracellular autoxidation, the oxidation process could closely relate to the intracellular production of ROS in B16F10 melanoma cells. This speculation could agree with the experimental results that the increased intracellular amount of ROS in B16F10 cells having tyrosinase was higher than that of HaCaT cells having no tyrosinase (Figure 4).

The production of superoxide is enhanced by adding H_2_O_2_ and dopa to tyrosinase solution [36]. Moreover, H_2_O_2_ is reported to be generated in oxidization process from phenol derivative to quinone derivative by copper [37]. 2,3-Dihydroxybenzoic acid, which is a natural polyphenol whose structure is similar to that of RD-catechol, enhances oxidation reaction with copper in the presence of H_2_O_2_ [38].H_2_O_2_ is now well-known to be produced in the generating process of quinone by the oxidization of phenolic hydroxyl group of anthrahydroquinone on industrial scale process, which is called anthraquinone oxidation (AO) process [39]. The results of these reports agree with the speculation that H_2_O_2_ was generated in the oxidization process of RD in this study (Figure 12), and RD-quinone might be converted into RD-quinone radical (RD-quinone^∙^), through the conjugation of allylic C-H bonds with the *π*-bond (*π*-allylic conjugation) [40, 41]. Therefore, since divalent metal oxidizes to univalent metal with the oxidization of metalloprotein [23, 28–30], Cu (II) in tyrosinase was assumed to be oxidized to Cu(I) with generating hydrogen through the generating pathway of RD-quinone^∙^ as indicated in the following:

(1) Cu(II)-tyrosinase+RD ⟶Cu(I)-tyrosinase+RD-quinone∙+H+

Since the reaction of univalent metalloprotein with H_2_O_2_ induces the generation of OH^∙^ [23, 28–30], copper in tyrosinase was thought to be converted as indicated in the following:

(2) Cu(I)-tyrosinase+H2O2 ⟶Cu(II)-tyrosinase+OH∙+OH−

The formation of a *π*-allylic conjugation in phenolic compounds suggested that the conjugated system extends to the side chain of phenolic compounds, and electrons were able to move between the terminal oxygen atom of the side chain and the benzene ring of phenolic compounds. The distance between the terminal oxygen atom of the side chain and the benzene ring of RD or RK was speculated to be comparable to that of L-tyrosine, indicating that the moving distance of electrons in RD or RK might be the same as that in L-tyrosine, relating to a melanocytes-specific cytotoxicity induced by RD or RK.

RD is approved to be used as a skin-brightening ingredient in quasidrug category in Japan. Therefore, many safety evaluation tests for RD have been carried out, and the safety of RD is ensured at the same safety level as that of medicine. However, RD has induced a strong adverse effect, leukoderma, in 2013. There is a question why RD has passed many safety tests. This study confirmed that RD induced a melanocytes-specific cytotoxicity and increased an intracellular ROS level in B16F10 cells. Although three-dimensional cultured skin models are used in safety evaluation tests, there are no safety evaluation tests using melanocytes. Therefore, for improving the safety of quasidrug or cosmetic ingredient, safety evaluation tests, which investigate whether a test compound shows a melanocytes-specific cytotoxicity and increases an intracellular ROS level in melanocytes, are suggested to be necessary. The authors hope that this study's results could contribute to the establishments of these safety evaluation tests.

## 5. Conclusions

RD increased the intracellular H_2_O_2_ level in B16F10 cells. Since the structure of RD is similar to that of L-tyrosine, RD is thought to react with copper in tyrosinase. Moreover, the results of reverse-phase HPLC analysis suggested that RD was oxidized to a quinone derivative through catechol and semiquinone derivatives by tyrosinase. Therefore, H_2_O_2_ was speculated to be generated in the oxidization process to quinone derivative from RD in B16F10 cells. Furthermore, ESR spectroscopy in this study confirmed that OH^∙^, which is known to show a high cytotoxicity, was generated by the reaction of tyrosinase and H_2_O_2_, indicating that reaction of copper in tyrosinase and H_2_O_2_ might induce Fenton reaction simultaneously. This study proposed that the RD-induced melanocytes-specific cytotoxicity could be caused possibly by OH^∙^ generated by the reaction of copper in tyrosinase and H_2_O_2_, and this reaction might be one of the mechanisms for explaining RD-induced leukoderma.
